# Establishing a Structured Hypospadias Biobank Cohort for Integrated Research: Methodology, Comprehensive Database Integration, and Phenotyping

**DOI:** 10.3390/diagnostics15050561

**Published:** 2025-02-26

**Authors:** Tariq O. Abbas, Kholoud Al-Shafai, Asma Jamil, Maraeh Mancha, Amina Azzah, Seem Arar, Sushine Kumar, Alia Al Massih, Rafah Mackeh, Sara Tomei, Luis R. Saraiva

**Affiliations:** 1 Urology Division, Sidra Medicine, Doha P.O. Box 26999, Qatar; 2 Weil Cornel Medicine-Qatar, Cornell University, Doha P.O. Box 24144, Qatar; 3 College of Medicine, Qatar University, Doha P.O. Box 2713, Qatar; 4 Division of Translational Medicine, Research Branch, Sidra Medicine, Doha P.O. Box 26999, Qatarlsaraiva@sidra.org (L.R.S.); 5 Research Department, Sidra Medicine, Doha P.O. Box 26999, Qatar; 6 Perioperative Services, Sidra Medicine, Doha P.O. Box 26999, Qatar; 7 Department of Comparative Medicine, Yale University School of Medicine, New Haven, CT 06510, USA; 8 College of Health & Life Sciences, Hamad Bin Khalifa University, Doha P.O. Box 34110, Qatar

**Keywords:** biobank cohort, hypospadias, database, phenotyping, pediatric urology, sample collection, genomics, data integration

## Abstract

**Background/Objectives**: Hypospadias, a common congenital anomaly in males, presents significant challenges in diagnosis, management, and long-term care. Despite its prevalence, research into the condition has been hampered by the lack of integrated biobank cohorts linking clinical, phenotypic, and surgical data with biological samples. This study aimed to establish the Hypospadias Biobank Cohort (HBC), a comprehensive resource designed to advance the understanding of hypospadias etiology and improve patient outcomes. **Methods**: The HBC was developed using a multi-phase approach, enrolling participants from specialized clinics between April 2022 and September 2024. Biological samples (blood and tissue) were collected under standardized protocols following informed consent. Detailed clinical data, including hypospadias severity, associated anomalies, and surgical outcomes, were systematically recorded and integrated into a robust database to support translational research. **Results**: The cohort included a diverse group of patients with varying severity of hypospadias, many of whom also presented with associated anomalies. Surgical outcomes were tracked, revealing important correlations between severity and postoperative complications. Preliminary biological analyses identified potential biomarkers associated with hypospadias severity and recovery. The full details of these results will be presented in a separate publication. The comprehensive database is continuously updated with longitudinal follow-up data, supporting future translational research. **Conclusions**: The Hypospadias Biobank Cohort represents a groundbreaking resource for translational research, offering unprecedented insights into the clinical and phenotypic spectrum of hypospadias. By enabling the refinement of classification systems and the development of evidence-based surgical techniques, the HBC has the potential to transform the management of this congenital condition. Ongoing research leveraging the HBC will further unravel the complex interplay among clinical presentation, surgical interventions, and patient outcomes, paving the way for personalized care strategies and improved long-term results.

## 1. Introduction

Hypospadias is one of the most common congenital anomalies affecting the male urethra, with an estimated incidence of 1 in ~200–300 live births. This condition is characterized by an abnormal location of the urethral opening along the ventral aspect of the penis, which can occur at various sites, including the glans, shaft, or perineum. Hypospadias severity is classified into three primary types: distal (glanular), midshaft, and proximal, each of which presents unique clinical and surgical challenges. 

The etiology of hypospadias remains unclear, although several key factors have been implicated to date. Genetic predisposition is thought to play a significant role, with familial patterns observed in some cases. Genome-wide association studies have identified additional loci, including DGKK variants, that are linked with hypospadias, although these findings only explain <10% of the associated genetic factors [1,2]. Environmental influences, such as exposure to endocrine disruptors, maternal diabetes, and advanced paternal age, have also been associated with increased risk [3]. Despite these associations, the precise mechanisms underlying hypospadias development are still not fully understood, highlighting the need for more extensive research.

The current management of hypospadias typically involves surgical intervention to correct the urethral opening and restore normal urinary function and appearance. Surgical techniques vary based on the type and severity of the condition. While many patients achieve satisfactory outcomes, complications such as stricture, fistula formation, and dissatisfaction with cosmetic results are major concerns. These challenges underscore the importance of understanding the multifactorial origins of hypospadias to inform both preventive strategies and surgical approaches. 

The absence of a centralized biobank cohort for hypospadias research has hindered progress in this area [4]. Existing studies often lack comprehensive datasets that integrate clinical, genetic, and phenotypic information, making it difficult to draw meaningful conclusions about risk factors, outcomes, and best practices [5]. A high-quality biobank cohort could, therefore, offer valuable resources for researchers and clinicians, facilitating the collection and analysis of integrated data to better unravel the complexity of hypospadias [4]. 

In this report, we describe the establishment of the Hypospadias Biobank Cohort (HBC), focusing on the systematic collection of biological samples alongside comprehensive clinical, ‘omic’, and phenotypic data. By integrating these diverse data types, we hope to foster a better understanding of hypospadias etiology, improve surgical outcomes, and ultimately enhance patient care. The HBC will also enable future studies of genetic and environmental influences that advance the broader field of congenital anomaly research.

## 2. Materials and Methods

### 2.1. Biobank Cohort Design and Development

A multi-phase approach was used to create the HBC, starting with obtaining informed consent and collecting biological samples (blood and tissue) following standardized protocols. Detailed clinical data, including hypospadias severity, associated anomalies, and surgical outcomes, were carefully recorded. These data were then integrated into a comprehensive database, designed to support future research. The protocol was developed in accordance with the SPIRIT (Standard Protocol Items: Recommendations for Interventional Trials) 2013 checklist for documenting a protocol study [6].

### 2.2. Recruitment Strategy

#### 2.2.1. Inclusion and Exclusion Criteria

The biobank cohort participants were selected using specific inclusion criteria to ensure a comprehensive representation of hypospadias diagnoses, including distal, midshaft, and proximal types. Our aim was to capture the full range of phenotypic variability to allow for a better understanding of the hypospadias spectrum and associated genetic factors [7]. The exclusion criteria included individuals with syndromic conditions, or any known genetic disorders that could confound the results of future analyses [8]. 

#### 2.2.2. Ethical Considerations and Consent Process

Participants and their guardians were approached for informed consent during routine clinical visits (Sidra IRB protocol number: 1866246). The consent process was designed to be clear and comprehensive, ensuring that potential participants understood the purpose of the biobank cohort, the sample types collected, and how their data would be used. In pediatric cases, consent was obtained from parents or legal guardians, with assent sought from older children (>12 years) when appropriate. Ethical considerations included patient confidentiality, the right to withdraw from the study at any time, and assurance that participation would not affect quality of care [9]. Eligible participants who could not provide informed consent/assent due to medical reasons or language barriers were excluded. Recruitment into the HBC was conducted via the Pediatric Urology Division of Sidra Medicine, which is a state-of-the-art healthcare and research institution in Qatar specializing in women and children’s health services. Our strategy included active recruitment, with clinicians informing eligible patients and families about the study during their appointments. We initiated a multi-site partnership and invited involvement from additional centers to expand the participant pool, hence augmenting biobank cohort diversity and significance. Recruitment commenced on 20 April 2022, and a total of 87 people were registered by September 2024.

### 2.3. Sample Collection and Processing

#### 2.3.1. Types of Biological Specimen Collected

**Figure 1:**
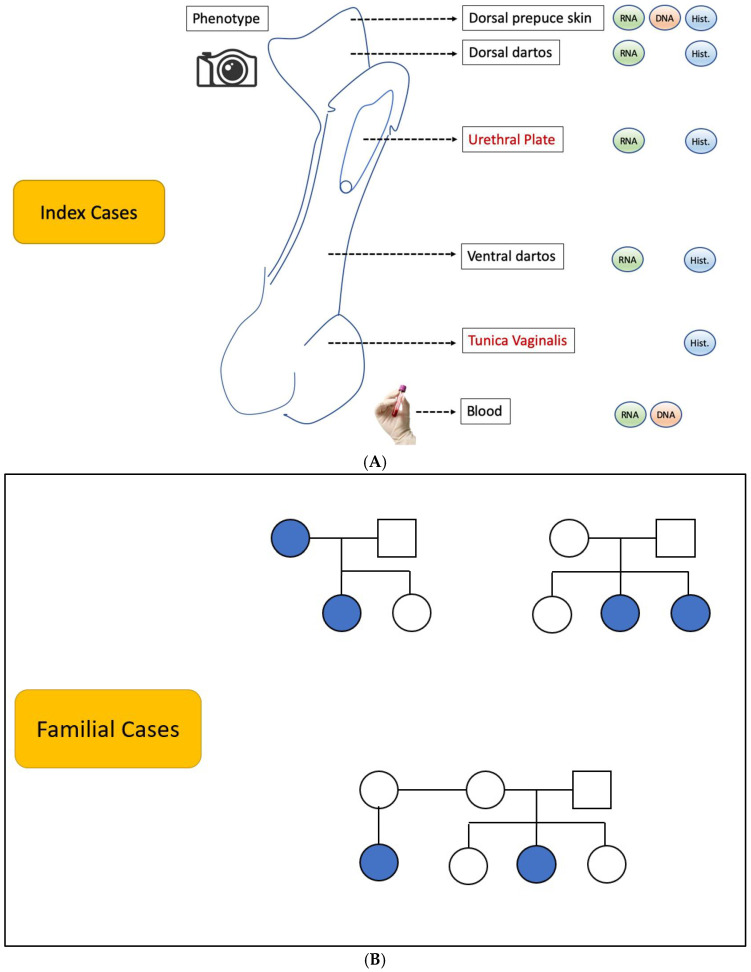
(**A**) Tissue specimens collected intraoperatively from each index patient. (**B**) Example familial cases enrolled for blood collection via the clinic (families in which more than one male member is affected by hypospadias).

A variety of biological samples were collected from the biobank cohort to support multifaceted research projects [9]. These included specimens, as detailed in Figure 1A,B: tissue samples were obtained during surgical procedures for histopathological examination and molecular studies, such as RNA sequencing (RNA-seq), DNA methylation/epigenetic profiling, and functional studies; blood samples were obtained for DNA extraction.

#### 2.3.2. Sample Handling Protocols

All samples were handled according to strict protocols to ensure optimal tissue integrity and data reliability. Upon collection, the samples were registered onto the Sidra Laboratory Management Information System (LIMS). The samples received were labeled with de-identified unique codes to maintain participant confidentiality. The samples were placed into appropriate containers and transported to the laboratory within 1 h of collection, where they were immediately processed or stored under optimal conditions.

#### 2.3.3. Storage Conditions and Labeling Systems

**Figure 2:**
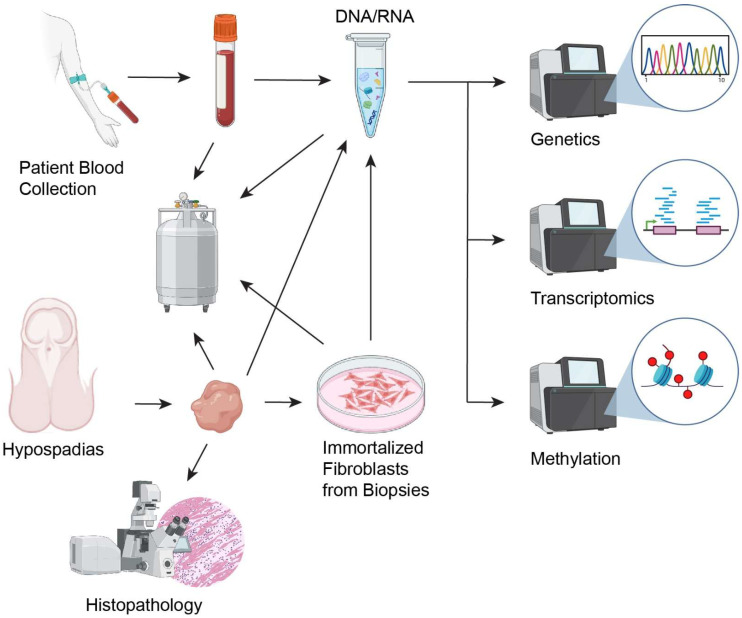
Flowchart diagram of sample collection and analysis protocol.

Biological samples were stored at specific temperatures based on their type (Figure 2):

- Tissue Samples: Fixed in formalin for histological analysis.
  - RNA Analysis: Two tissue samples per subject were obtained for RNA profiling. Tissue specimens were submerged in a ~5-fold volume of RNAlater^®^ solution (Thermo Fisher Scientific, Waltham, MA, USA) at room temperature to stabilize the RNA and eliminate the need for immediate freezing. The samples were then kept at 4 °C for up to 5 days before long-term storage at −80 °C [10].
  - DNA Analysis: Two tissue samples per subject were collected for DNA analysis. The samples were submerged in DNA preservative AllProtect Tissue Reagent (QIAGEN, Valencia, CA, USA) at room temperature to eliminate the need for immediate freezing [11]. The samples were then kept at 4 °C for up to 5 days before long-term storage at −80 °C.
  - Primary Fibroblast Generation: The epidermis was removed from a 0.5 × 0.5 cm^2^ sample of preputial skin, which was then placed in Dulbecco’s Modified Eagle Medium (DMEM) supplemented with 10% fetal bovine serum and 1× penicillin/streptomycin for transfer to the lab and subsequent processing.
  - Histological Examination: The remainder of each surgical specimen was routinely processed by fixation in formalin to preserve tissue structure for subsequent histological studies.
- Blood Samples: Peripheral blood (total volume of 4–5 mL) was collected into EDTA-containing test tubes and kept in the fridge until aliquoting into 2 mL cryotubes for long-term storage at −80 °C. Each EDTA tube yielded three biological samples: 2 mL of plasma, 2 mL of precipitate, and the buffy coat remainder. For proteomic analysis, Hulmes et al. demonstrated that plasma sample quality can be enhanced by adding a protease inhibitor cocktail directly to collection tubes prior to phlebotomy, centrifuging samples with in 1 h of blood draw, and then immediately snap-freezing aliquots for storage at −70 °C [12]. EDTA anticoagulation is regarded as optimal for plasma proteome stability and coverage [13].

#### 2.3.4. Processing Steps for Long-Term Storage

The following protocols were implemented for long-term sample storage and future analyses: for DNA/RNA Extraction, samples underwent standard extraction protocols using commercial kits to isolate high-quality nucleic acids for downstream applications. Regular assessments of sample integrity and viability were conducted, ensuring that only high-quality samples were retained in the biobank cohort.

#### 2.3.5. Integration with Comprehensive Database

##### Database Design and Infrastructure

The comprehensive HBC database was designed to facilitate efficient storage, retrieval, and analysis of clinical and phenotypic data. The architecture employs a relational database management system (RDBMS) using REDCap as the primary platform. REDCap provides a secure web-based interface that allows for customizable data collection and management, making it an ideal solution for HBC needs.

**Figure 3:**
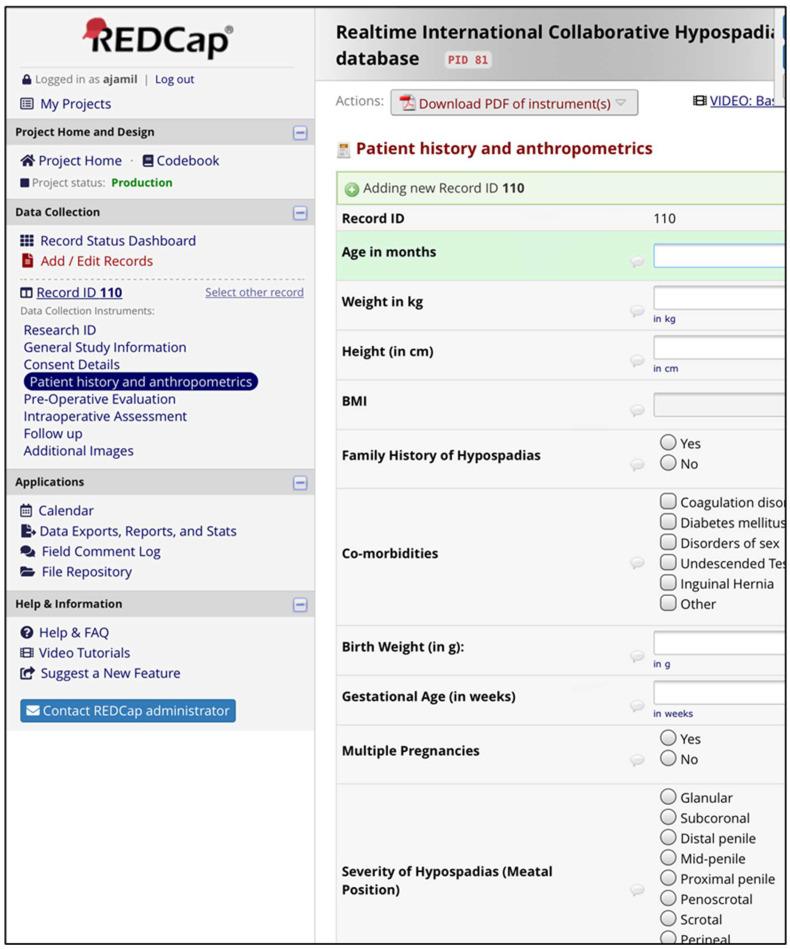

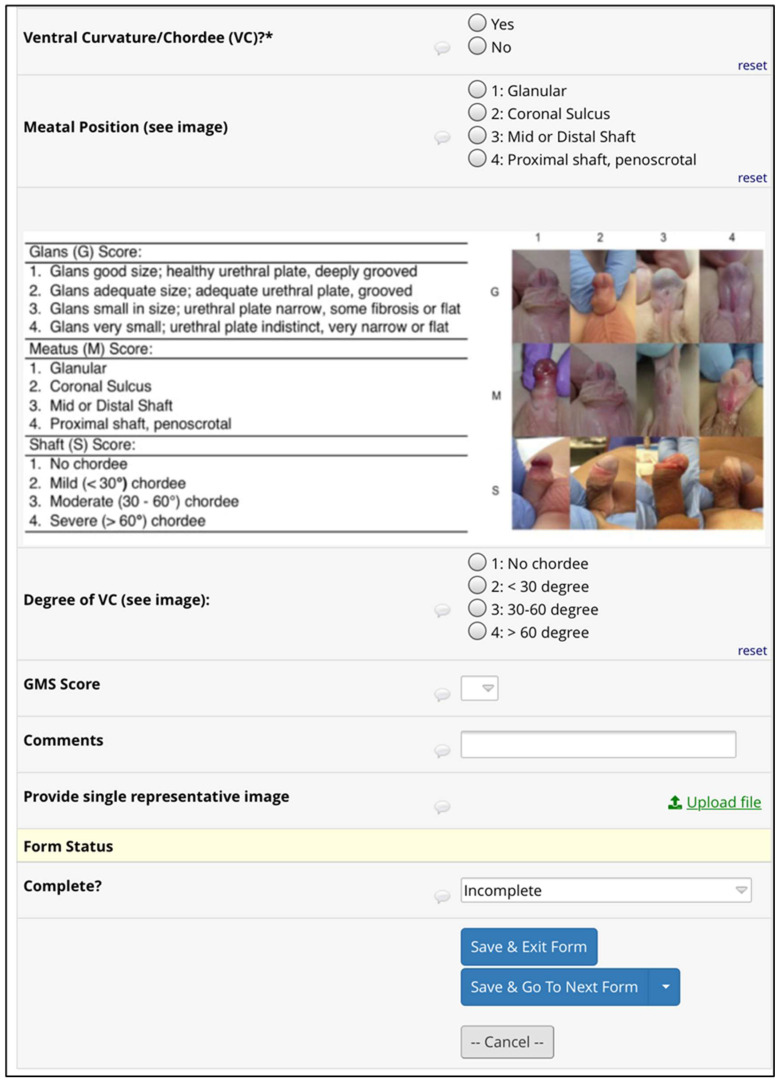

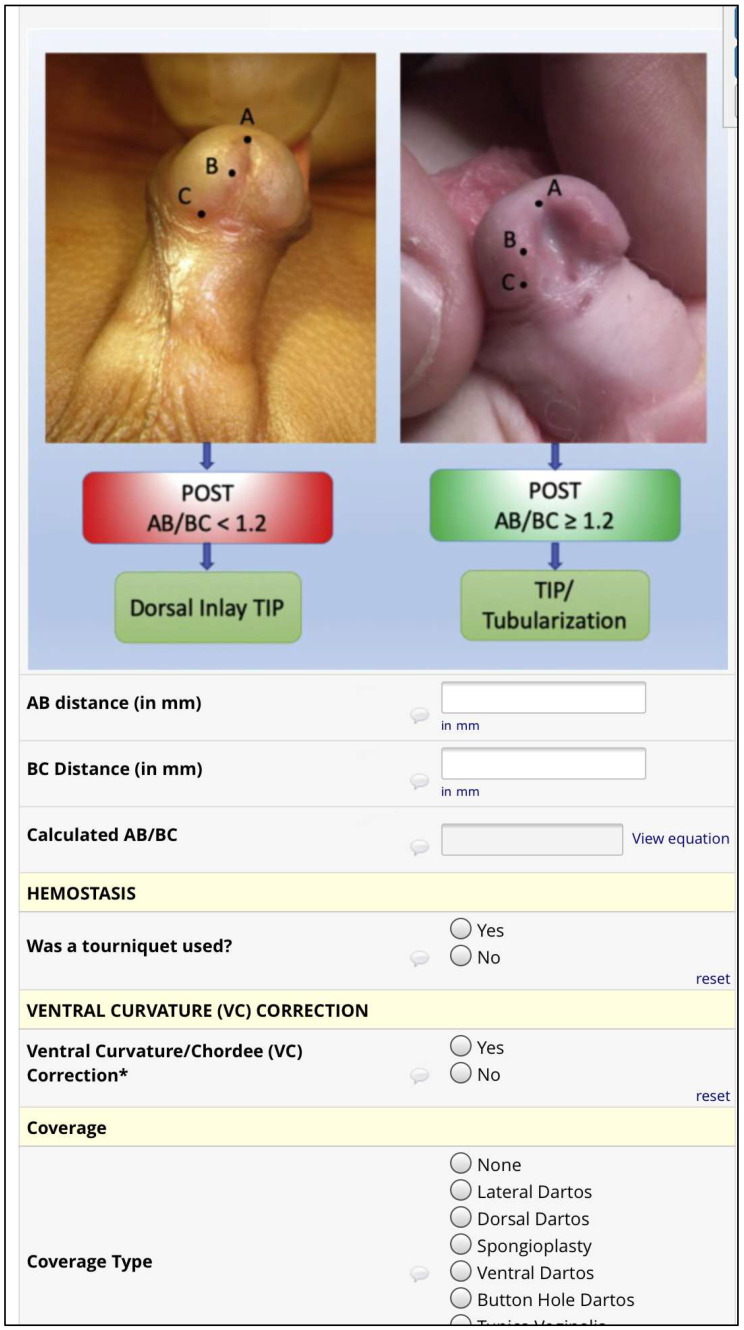
Example screenshots from the HBC database.

The database is structured to encompass the following data categories:

1. Patient Demographics: Information such as age, sex, ethnicity, and socioeconomic status.
2. Clinical Variables: Detailed medical history, including hypospadias type and severity, associated comorbidities, and family history.
3. Treatment Data: Information regarding surgical procedures, postoperative care, and any complications encountered.
4. Outcomes: Data on long-term outcomes, including functional results, satisfaction scores, and any further interventions required (Figure 3)

This structured approach permits easy querying and data analysis, facilitating comprehensive research into the factors that influence hypospadias and its management.

##### Data Linkage

To ensure robust integration of HBC biological samples with clinical information, a systematic data linkage approach was implemented. Each biological sample collected was assigned a unique identifier that corresponds to the participant’s record in the comprehensive database. Each sample was then labelled with the letters HBCB (for hypospadias biobank cohort) and four digits forming a number from 0001 to 9999 in numerical sequence. These identifiers allow for seamless tracking and the association of biological data with clinical variables. Training sessions on data entry procedures and terminology were conducted to minimize discrepancies and enhance uniformity of the approach by clinical staff. Regular audits and quality checks will be performed to assess data completeness and accuracy going forward. By leveraging the capabilities of REDCap for data management and implementing stringent linkage and harmonization processes, robust procedures are in place to ensure that the biobank cohort yields high-quality, reliable data that can drive meaningful research on hypospadias and its associated clinical challenges.

##### Data Input and Quality Control

Data were entered using a standardized electronic data capture system, which minimizes transcription errors. All clinical data, genetic information, and phenotypic details were input directly into the database by trained personnel. Each data entry underwent a two-step validation process. Initially, the data were checked for completeness and consistency at the time of entry. Subsequent periodic audits were conducted to cross-verify entries against source documents, ensuring alignment with clinical records.

##### Data Management and Accessibility

Researchers and clinicians can access HBC records based on role-specific permissions. A tiered access model is in place, where sensitive data are restricted to authorized personnel only. Access requests are reviewed by a governance committee to ensure compliance with ethical standards. The database will be updated regularly to incorporate new information from ongoing research activities and follow-up studies. A dedicated data management team is responsible for routine maintenance, ensuring that the database remains current and functional. Scheduled backups are performed to prevent data loss. To safeguard sensitive patient information, the biobank cohort employs robust security measures, including encryption for both data storage and transmission purposes. Access controls are enforced through user authentication protocols, and all interactions with the database are logged for audit purposes.

### 2.4. Phenotyping Methodology

#### 2.4.1. Phenotypic Data Collection

A. Phenotypic Variables Recorded: The biobank cohort captures a range of phenotypic data related to hypospadias anatomical variables, including the following:
  - Location of the urethral opening [14];
  - Plate objective scoring tool (POST) [14,15,16];
  - Urethral defect ratio (UDR) [7];
  - Penile curvature [17].
B. Key Clinical Variables: The essential clinical variables are basic patient demographics (age, ethnicity, etc.) and associated congenital anomalies. The measurement techniques included clinical assessments by trained urologists, photographic documentation of anatomical features, and standardized questionnaires for evaluating functionality (like uroflowmetry). Cosmetic outcomes were graded using the Pediatric Penile Perception Score (PPPS) [18] and Hypospadias Objective Penile Evaluation (HOPE) [19]. Each variable was recorded in the electronic database following strict protocols to ensure consistency and accuracy.
C. Surgical Outcomes: The outcomes include complication rates and the need for reoperation, as well as developmental follow-ups (assessment of urinary function, psychosocial outcomes, and quality of life). Data were collected at multiple time points to provide a longitudinal overview of patient progress.

#### 2.4.2. Standardization and Classification

To ensure comparability of phenotypic data, standardized protocols were employed across all participating centers. These included specific guidelines for data collection, anatomical assessments, and follow-up evaluations. Regular training sessions and calibration exercises were conducted to align practices between clinical staff and enhance inter-rater reliability. One doctor (T.A.) was responsible for the collection, and then trained 2 team members before the start of the next phase of the project. A standardized scoring system was used to grade hypospadias severity, which incorporates factors such as the urethral opening location, penile curvature, POST, UDR, and associated anomalies. Surgical success was classified using established criteria that consider postoperative complications and functional outcomes, while long-term patient outcomes were evaluated using validated tools that assess urinary function and psychosocial aspects. These classification systems facilitate consistent data interpretation and enhance the ability to conduct comparative studies.

#### 2.4.3. Integration with Genetic and Molecular Data

Phenotypic data will be systematically linked with genetic and molecular data collected from the biobank cohort, enabling comprehensive studies on genotype–phenotype correlations. Each participant’s phenotypic information, including surgical outcomes and anatomical variables, will be associated with the results of genomic sequencing and other molecular analyses. This integration will facilitate the identification of specific gene variants and pathways associated with hypospadias severity and related clinical outcomes, thus enhancing our understanding of the fundamental biology underpinning this condition [20]. 

#### 2.4.4. Data Analysis and Research Applications

Advanced statistical methods, including genome-wide association studies (GWAS), phenome-wide association studies (PheWAS), RNA sequencing, and others, will be utilized to identify gene variants, expression profiles, and methylation patterns that are linked to specific phenotypic traits. Additionally, regression analyses will explore relationships between phenotypic characteristics and clinical outcomes. Multi-omic integration techniques will combine genomic, transcriptomic, and epigenomic data to provide a holistic view of the factors that influence hypospadias pathogenesis. Machine learning algorithms will be applied to analyze phenotypic data, identify patterns, and develop predictive models for improved patient stratification. The biobank cohort will be instrumental in exploring key research questions and identifying biomarkers of hypospadias severity and response to different treatments. By correlating genetic data with clinical phenotypes, researchers will gain new insights that can inform personalized treatment strategies and improve patient care.

#### 2.4.5. Research Applications and Collaborations

The hypospadias biobank cohort provides a valuable resource for a variety of research projects, including studies on genetic risk factors, investigations into the effectiveness of different surgical approaches, and long-term clinical outcome studies assessing urinary function and psychosocial development. Additionally, the integration of phenotypic, genetic, ‘omic’, and functional data will facilitate important research into genotype–phenotype correlations, potentially leading to the identification of the biomarkers of severity and treatment response [20]. The HBC encourages collaboration with other biobank cohorts and research institutions worldwide to enhance global reach and impact (including via large-scale studies on diverse patient populations and/or cross-institutional data analyses). Collaborative efforts may also include multi-national research initiatives that aim to understand hypospadias epidemiology and improve management strategies across different healthcare systems.

### 2.5. Ethical, Legal, and Social Issues

#### 2.5.1. Patient Consent and Ethical Approval

The biobank cohort protocol underwent thorough review and approval by an institutional ethics committee to ensure compliance with ethical standards and guidelines. The review process assessed the study’s objectives, methodologies, and potential risks to participants. Informed consent procedures were specifically tailored for children and their families. Consent forms were designed to be age-appropriate, providing clear explanations of the study purpose, procedures, and potential risks. Parents or guardians were actively involved in the consent process, ensuring that they understood the implications of their child’s participation and had the opportunity to ask questions.

#### 2.5.2. Data Privacy and Security

To ensure data privacy, the biobank cohort employs pseudonymization techniques that separate personal identifiers from the research data. This minimizes the risk of re-identification [21]. The data are stored securely in encrypted databases with restricted access to authorized personnel only. The HBC operates in compliance with national and international biobank cohort regulations, including adherence to data protection laws such as the General Data Protection Regulation (GDPR). Regular audits are conducted to ensure ongoing compliance with legal and ethical standards.

#### 2.5.3. Public Engagement

Participants are kept informed about ongoing research through newsletters, information sessions, and direct communication. This transparency helps maintain trust and encourages continued involvement in the biobank cohort’s research initiatives.

### 2.6. Challenges and Future Directions

#### 2.6.1. Challenges Encountered

Several logistical, technical, and ethical challenges were encountered during the biobank cohort setup: A significant challenge was participant recruitment, particularly given the sensitive nature of hypospadias. Engaging families and ensuring willingness to participate required extensive outreach efforts and education about the HBC’s purpose and potential benefits. Navigating the ethical landscape associated with biobank cohorting, especially regarding informed consent for minors, required careful attention. Tailoring consent processes to address the unique concerns of families was vital in building trust and facilitating participation.

#### 2.6.2. Future Expansion

Looking ahead, plans are in place to expand the biobank cohort’s scope. Future efforts will focus on including more diverse patient populations to enhance the generalizability of research findings. Collaborations with various healthcare institutions across geographical regions are expected to facilitate this expansion. The biobank cohort aims to incorporate longitudinal follow-up data to track patient outcomes over time. Additionally, we plan to integrate environmental data, as well as new anatomical variables, diagnostic approaches, and artificial intelligence tools [22,23], which may provide further insights into the external factors that influence hypospadias development and management. As biobank cohorting technologies evolve, the HBC will adapt to incorporate cutting-edge techniques, such as single-cell sequencing data and organoid development. These advancements will allow for more detailed investigations into the cellular and molecular basis of hypospadias.

## 3. Results

The cohort successfully enrolled a diverse group of participants, including male infants, children, and adolescents diagnosed with varying degrees of hypospadias, ranging from mild to more severe forms. Many participants presented with associated anomalies, such as chordee or undescended testes. The cohort represented a wide range of ethnic backgrounds, which is essential for identifying potential genetic variations across different populations. Detailed results will be described in a separate publication, as the current focus is on the description of the protocol. The comprehensive database is continuously updated with longitudinal follow-up data to support future translational research.

### 3.1. Clinical Data Analysis

The clinical data collected as part of the HBC included detailed medical histories, physical examinations, and information about surgical interventions. A range of hypospadias severity was observed, with a majority of participants presenting with distal hypospadias, followed by moderate (midshaft), and more severe proximal hypospadias, the latter often requiring complex surgical correction. Some patients with proximal hypospadias presented with additional malformations, such as undescended testes, inguinal hernias, or other genital anomalies, which highlights the importance of a holistic approach in managing these patients. A subset of patients had undergone corrective surgery, and the post-surgical outcomes, including functional, cosmetic results, and complications (e.g., fistula formation, meatal stenosis), were recorded. Preliminary analysis indicates a higher rate of successful outcomes in patients with distal hypospadias, with fewer complications compared to those with more proximal defects. Additionally, some participants had co-occurring congenital abnormalities, such as cryptorchidism or inguinal hernias, emphasizing the need for comprehensive evaluation in hypospadias cases. Detailed findings will be described in a separate publication, as the current focus is on the protocol description. The database is continuously updated with follow-up data to support ongoing research.

### 3.2. Genetic Data and Associations

Genetic testing, including whole-genome sequencing and targeted gene panels, revealed promising insights into the genetic underpinnings of hypospadias. Early analyses have led to the identification of several candidate genes and genetic loci that may contribute to the development of hypospadias.

### 3.3. Phenotypic Data

Phenotypic data were collected using standardized protocols to classify hypospadias severity and type, focusing on the meatal location, urethral length, penile length, chordee, and testicular anomalies. The meatal position varied, with distal cases having a meatus near the tip of the penis and proximal cases closer to the scrotum. Shorter urethras were more common in severe cases, influencing surgical outcomes. The majority of patients with proximal hypospadias had chordee, requiring surgical correction, and penile length was systematically recorded to guide treatment and assess post-operative results. Additionally, some of the patients had undescended testes, often linked to more severe hypospadias, necessitating close monitoring for testicular descent and long-term fertility and hormone function. Detailed findings will be described in a separate publication, as the current focus is on the protocol description.

### 3.4. Data Integration and Early Insights

The integrated clinical, genetic, and phenotypic data have already begun to provide valuable insights into the complex nature of hypospadias. The correlation of genetic markers with phenotypic variations (such as severity and co-occurring anomalies) is helping to identify potential genetic risk factors and improve the classification of hypospadias based on underlying genetic profiles. The initial data suggest that more severe forms of hypospadias may be associated with specific genetic variants, while milder forms may be influenced by a combination of genetic and environmental factors. These findings have the potential to refine the current understanding of hypospadias classification and may ultimately guide more personalized treatment approaches in the future.

## 4. Discussion

The Hypospadias Biobank Cohort provides a critical framework for advancing the understanding of hypospadias, offering a comprehensive approach that integrates clinical, phenotypic, and biological data. By following standardized protocols, the cohort ensures consistency and quality, supporting future research on the genetic, environmental, and clinical factors influencing hypospadias. While the detailed results of patient outcomes and analyses will be separately described in another report, this biobank will serve as a valuable resource for refining classification systems, guiding surgical interventions, and informing long-term management strategies. The establishment of a high-quality biobank cohort dedicated to hypospadias research will be a pivotal step toward improved understanding of this complex congenital anomaly. This discussion section explores the implications of the HBC’s methodologies, potential contributions to future research, ethical considerations, and the anticipated challenges moving forward.

The HBC’s structured approach to data capture and linkage allows for the seamless integration of clinical and biological information. By assigning unique identifiers to patient specimens, we ensure robust tracking and association with corresponding clinical records. This meticulous process enhances data integrity and allows for nuanced analysis of the multifactorial aspects of hypospadias. Comprehensive phenotypic data collection—including anatomical variables, surgical outcomes, and psychosocial assessments—facilitates a holistic view of patient experiences. The use of standardized scoring systems and validation steps significantly increases the reliability of the data collected. These methods not only enhance inter-rater reliability among clinical staff but also support the reproducibility of research findings, which is crucial for the advancement of clinical practices in hypospadias management [24].

The biobank cohort provides a rich resource for exploring key questions in hypospadias research. By linking phenotypic data with genetic profiles, we can investigate genotype–phenotype correlations that may reveal underlying biological mechanisms that determine hypospadias severity [25]. This integration could also lead to the identification of novel biomarkers of severity and treatment responses, ultimately informing personalized treatment strategies.

Furthermore, the potential for long-term follow-up studies allows us to assess both patient trajectory and developmental outcomes in hypospadias cases. The incorporation of environmental factors and new diagnostic tools will enable researchers to explore the influence of multiple additional variables. By engaging in multi-omic integration, we aim to provide a comprehensive understanding of the interplay between genetic risk factors and environmental influences on hypospadias [26,27].

The ethical framework established for the HBC is designed to ensure participant safety and data integrity. The informed consent process, tailored for children and their families, emphasizes transparency and education, fostering trust between researchers and participants. It is imperative that we continue to review the biobank cohort’s ethical landscape, especially concerning the involvement of minors and consenting processes, as we continue to expand the scope of our research. Data privacy remains a cornerstone of our ethical commitment. By employing pseudonymization techniques and adhering to stringent data protection regulations, such as GDPR, we minimize the risk of re-identification and uphold the confidentiality of participant information [28]. Ongoing audits will ensure compliance with legal and ethical standards, reinforcing our commitment to ethical research practices.

While the biobank cohort holds immense potential, we anticipate several challenges as this initiative progresses. Recruitment remains a significant hurdle, especially given the sensitive nature of hypospadias. Engaging families in a manner that emphasizes the benefits of participation requires ongoing outreach efforts and clear communication about study goals and potential impacts. Building relationships with communities and healthcare providers will be crucial to overcome these recruitment barriers. Additionally, the integration of diverse patient populations is vital to enhance the generalizability of findings derived from HBC resources. Collaborations with healthcare institutions across different regions will be essential for reaching a broader demographic. This will not only enrich HBC datasets but also provide new insight into the epidemiology of hypospadias across different demographics.

Looking ahead, the biobank cohort is poised for expansion in several key areas. The inclusion of longitudinal follow-up data will allow for a deeper understanding of patient outcomes over time. Moreover, exploring environmental factors and integrating advanced technologies such as artificial intelligence tools can significantly enhance our research capabilities. As we adapt to the evolving landscape of biobank cohorting, incorporating cutting-edge techniques, such as single-cell sequencing, will provide further novel insight into the cellular mechanisms underlying hypospadias.

## 5. Conclusions

In conclusion, the biobank cohort represents an important new platform for hypospadias research and analysis of the genetic, phenotypic, and environmental factors that impact this condition. By fostering collaborative efforts and maintaining rigorous ethical standards, we are now positioned to unlock new insights that will ultimately lead to more effective management strategies and improved patient outcomes. As we embark on this journey, we remain committed to the principles of transparency, inclusivity, and scientific rigor, ensuring that our research is both impactful and ethical.

## Data Availability

The datasets generated and analyzed during the current study are not publicly available due to participant confidentiality and institutional data-sharing policies. However, de-identified data may be made available upon reasonable request to the corresponding author, subject to approval from the relevant ethics committee and compliance with institutional regulations.

## Abbreviations

The following abbreviations are used in this manuscript:

HBC	Hypospadias Biobank Cohort
DGKK	Diacylglycerol kinase kappa
SPIRIT	Standard Protocol Items: Recommendations for Interventional Trials
RNA	Ribonucleic acid
DNA	Deoxyribonucleic acid
LIMS	Laboratory Management Information System
DMEM	Dulbecco’s Modified Eagle Medium
REDCap	Research Electronic Data Capture
RDBMS	Relational Database Management System
HBCB	Hypospadias Biobank Cohort
POST	Plate Objective Scoring Tool
UDR	Urethral defect ratio
PPPS	Pediatric Penile Perception Score
HOPE	Hypospadias Objective Penile Evaluation
GWAS	Genome-wide association study
PheWAS	Phenome-wide association Study
GDPR	General Data Protection Regulation
